# Designing the *LAGOM* burnout prevention program: a collaborative approach with healthcare professionals using intervention mapping

**DOI:** 10.1186/s12909-025-07943-9

**Published:** 2025-09-16

**Authors:** Julia Katharina Schiele, Marleen Schröter, Julia Berschick, Melanie Steinmetz, Martin Bogdanski, Sandra Jankow, Wiebke Stritter, Christian S. Kessler, Georg Seifert, Anna K. Koch

**Affiliations:** 1 Charité Competence Center for Traditional and Integrative Medicine (CCCTIM), Charité – Universitätsmedizin Berlin, Corporate Member of Freie Universität Berlin, Humboldt-Universität zu Berlin, Augustenburger Platz 1, 13353 Berlin, Germany; 2 Department of Internal Medicine and Nature-Based Therapies, Immanuel Hospital Berlin, 14109 Berlin, Germany

**Keywords:** Intervention development, Healthcare professionals, Burnout, Intervention mapping, Prevention

## Abstract

**Background:**

Burnout is a global challenge and healthcare professionals are especially at risk. This paper outlines the development of a tailored, evidence-based, theory-driven intervention designed to prevent burnout among hospital-based healthcare professionals (*LAGOM* program). During development, particular attention was paid to cooperation and constant feedback loops with various stakeholders, especially with the participants of the intervention.

**Methods:**

The *LAGOM* project has been taking place at Charité - Universitätsmedizin Berlin and the Immanuel Hospital, Berlin since 2022. To develop, implement and evaluate the burnout prevention program, we conducted two reviews, interviews, focus groups and workshops. Central to the development process was the application of an Intervention Mapping (IM) approach and the Precede/Proceed logic model, which provided a structured, stepwise framework for translating needs assessment findings into targeted intervention strategies by following its six iterative steps. In addition to the multi-professional project team, various advisory boards, including the management of the two hospitals, were involved in the project. The healthcare professionals for whom the intervention was developed were actively involved in every phase of program development.

**Results:**

*LAGOM* (“Long-term Health-Related Organizational Concepts with Mind-Body Medicine”) includes 9-week in-person and online sessions covering stress patterns, communication, work culture reflection, self-care, and relaxation and exercise practices based on mind-body medicine. Participation in those sessions is working time. With endorsement from the management board of both hospitals, *LAGOM* also aims to enhance the work environment within the hospital structures, combining structural and behavioral prevention identified in the IM-guided development process.

**Conclusions:**

This article details the scientific development process for a burnout prevention program guided by the IM approach, thereby illustrating how intervention development methods can be employed to improve the standards for reporting on intervention development. Employing best practice frameworks, evidence-based behavior change techniques and close collaboration with healthcare professionals enhances effectiveness of *LAGOM.*

**Trial registration:**

The feasibility study and pragmatic controlled trial accompanied to LAGOM were prospectively registered with the German Clinical Trials Register (DRKS00032014, registered 17th October 2023 and DRKS00034060, registered 31st May 2024).

**Supplementary Information:**

The online version contains supplementary material available at 10.1186/s12909-025-07943-9.

## Background

Burnout poses a global challenge to society, particularly impacting healthcare professionals and compromising patient safety, care quality, professionalism, and workplace well-being [1–9]. Without effective burnout prevention embedded in corporate health management, the healthcare system is at risk. Evidence suggests interventions should be easily accessible at work, combining individual and structural aspects [10–15]. Establishing healthier work conditions as a standard practice should include implementing programs for preventing burnout, yet executing these remains a challenge (e.g. low adherence) and is still insufficiently researched [16–20]. Many interventions are effective only in ideal conditions and not in daily clinical routine [20]. To address this, interventions must be contextually relevant and developed based on a detailed needs assessment, considering the work environment. The cooperation of those who will participate in the program is also very important in the development of the intervention. Compared to externally developed approaches, interventions that involve employees in the local work environment in the design and implementation could increase their sense of control and engagement, which should lead to an effective reduction in burnout [8].

The intervention development presented in the present paper addresses these challenges. The overall goal of the project was to develop, implement, and evaluate a tailored, evidence-based, theory-driven intervention for burnout prevention among healthcare professionals working in a hospital. As the intervention developed here is intended to include structural prevention aspects in addition to individual prevention, the work environment played a major role during the IM process. The participation of healthcare professionals in the entire project process, including intervention development, also played a major role. For intervention development, we applied Bartholomew’s Intervention Mapping Approach [21, 22] and Green and Kreuter’s Precede logic model [23] as a framework.

The here developed intervention (*LAGOM*) has already undergone feasibility testing [24] and is currently undergoing effectiveness evaluation within a pragmatic randomized controlled clinical trial [25]. This paper describes the intervention and its development in detail following the six steps of IM. This is meant to help replicate and further develop the intervention.

## Methods

**Fig. 1:**
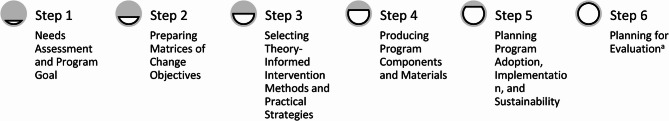
The six steps of the Intervention Mapping Approach [21, 26]. ^a^ Not the focus of this paper, see Koch, Schröter [25] for details

*LAGOM* is a Swedish word that describes the “golden mean”: Something is just right, not too much and not too little, the ideal balance. It is also an acronym for “**L**ong-term **A**pproach and **G**uidelines for **O**ccupational Mental Health with **M**ind-Body Medicine”. The development, implementation and evaluation of a custom tailored, evidence-based, theory-informed intervention inspired by mind-body-medicine (MBM) to prevent burnout was the overall project goal. Appropriate ethics votes were obtained for all steps of the project as needed (Ethics Committee of the Charité – Universitätsmedizin Berlin EA2/110/22, EA1/157/23, EA4/061/24). The feasibility study and pragmatic controlled trial accompanied to LAGOM were prospectively registered with the German Clinical Trials Register (DRKS00032014, registered 17th October 2023 and DRKS00034060, registered 31 st May 2024). We followed the six steps of the Intervention Mapping Approach to develop the intervention [21, 22], Fig. 1. The structure of this paper is based on the IM’s six-step approach.

### Project team

The core project team consisted of six people: A work- and occupational psychologist (AKK) with several years of expertise in study planning, biometrics and project design. Two PhD candidates: A psychologist (MS) with many years of study experience and MBM expertise in occupational prevention and a movement and mindfulness scientist (JB) with a qualitative focus. Further, a nutritionist (JS) with several years of expertise in MBM and guidance of MBM groups, an expert in electrophysiological measurements (MB) and an expert in strategic project management (MS) were part of the core project team. The core team worked closely together throughout the project with daily meetings. For the first three steps of the IM, the team worked together with the same tasks at the IM; from step 4 onwards, the tasks were then assigned with regard to the expertise (study design and planning; content conception of the *LAGOM* program; project management). The core team was supplemented by two physicians (one from the Immanuel Hospital (CSK), one from the Charité (GS)) who had both scientific expertise and actively worked at the respective sites with the professional groups for whom the course was to be developed and a psychologist (WS) with many years of experience in project implementation at the Charité. A one-hour project meeting was held once a week with the extended team to discuss the most important topics.

Additionally, various advisory boards were part of the project. Throughout the process, the individual steps were accompanied, supervised and critically reflected by those advisory boards. The expert advisory board consisted of up to 13 healthcare professionals, including nurses and medical doctors from various disciplines. An external research group comprising 25 researchers contributed additional scientific expertise to the program. A steering committee consisting of eight participants in key positions at executive level including the administrative director, hospital director, head of organizational development, chief executive officer, two nursing directors as well as a chief physician and the sustainability manager provided strategic oversight throughout the process. The expert advisory board as well as the steering committee included participants of each hospital sites. Two experts in the field of mind-body medicine were involved in the development process. Participants were recruited through stakeholder mapping and contacted via phone calls or emails based on their relevant institutional roles and expertise. The hospital staff for whom the intervention was developed was actively involved in all steps of the project, e.g. staff could engage in the development process without being in the expert advisory board through participation in focus groups. At any point in the intervention development, interested individuals had the opportunity to actively participate in one of these advisory boards, further supporting the intended user-centeredness. At regular meetings about every three months, all participants were informed about the current status of the project and their feedback was obtained.

#### Step 1: needs assessment and program goal

The first step of IM is to carry out a needs assessment to clarify the status quo regarding the health problem (in this case burnout) together with those affected (in this case healthcare professionals) and to define the program goal. This step identifies *personal determinants* and *environmental* as well as *behavioral factors* that contribute to burnout. This is based on the methodology of Green and Kreuter’s Precede logic model [23] to identify health outcomes and health problems for the intended program.

Our needs assessment is informed by five sources: [1] Six on-site work shadowings including five semi-structured interviews with healthcare professionals for whom the intervention was to be designed. The interview guide was developed for the study at hand (Supplement 5). Details of the interviews will be published in a subsequent manuscript [2]. An on-site think experiment (discussion workshop). This took place one afternoon on the hospital grounds of the Charité. Interested healthcare professionals could spontaneously drop by for a piece of cake and a cup of coffee and talk about questions in the context of the project and contribute their opinions and views (Manuscript in preparation) [3]. A scoping review summarizing current international workplace interventions aimed at reducing stress and/or preventing burnout among healthcare workers [27] [4]. A grey literature review with semi-structured interviews [12]. The integration and summarization of these five sources of information then results in the needs assessment and overall program goal.

#### Step 2: Preparing matrices of change objectives

Step 2 outlines the necessary changes to achieve the program goal defined in Step 1. All identified behavioral factors that contribute to burnout will be fully addressed in the program. Due to the large number of identified environmental factors and limited project resources, these must be prioritized and reduced to the most relevant ones. Hence, the relevance and changeability of the environmental factors is assessed by the project´s advisory boards (Supplement 1). On this basis, the environmental factors to be included in the program are selected through discussions within the project team. Then, each behavioral factor (e.g. healthcare professionals do not take regular breaks) is rewritten into desired behavioral outcomes (e.g. healthcare professionals take regular breaks). This is done by asking what needs to change so that certain behavioral risk factors lead to improved health outcomes. All behavior outcomes are then broken down into sub-goals, known as performance objectives (e.g. healthcare professional finds a suitable place without disruption to take a break). These were validated by the expert advisory board. Further, for each environmental performance objective, the environmental agents - i.e. the people who have the opportunity to initiate change due to their management position with regard to the relevant environmental condition - are identified. For the selected (changeable and relevant) environmental factors the team defines performance objectives. Those are later considered in the development of work environment strategies and further discussed with relevant stakeholders for implementation. The next step is to determine what people need in order to change their behavior, both on a personal level (*personal determinants* e.g. knowledge, attitude or skills) and on an external level (*external determinants* e.g. positive reinforcement, infrastructure or a certain leadership culture). To identify the personal and external determinants of the performance objectives, a working list of determinants was generated by brainstorming, reviewing the findings from the empirical literature using a comprehensive literature search, and testing the theories for additional constructs separate for each performance objective. The core project team then agreed on a list of the most relevant and changeable determinants (Supplement 2). By crossing performance objectives with determinants and writing change objectives, the final product of step 2 are the *matrices of change* with performance objective in the rows and determinants in the columns.

#### Step 3: selecting Theory-Informed intervention methods and practical strategies

In step 3, theory-informed methods that can influence change in personal or external determinants and conditions are identified (e.g. methods that increase the personal determinant “self-efficacy” or influence “attitude”). Based on this, theoretical methods (e.g. guided practice) and practical strategies (e.g. breathing exercise) for applying the methods to the intervention program are chosen. After generating evidence-based methods and strategies for all external and personal determinants the methods are rated regarding its fit within the population and clinical context as well as its relevance and changeability. Matrices of change, linking methods, strategies and possible program components with each identified change objective from step 2 are the final product of step 3. After that the project team generates program themes, components, scope, and sequence, order of component-delivery, setting and duration, messages, communication channels and finally selects or designs practical applications to deliver change methods (Supplement 3 and Supplement 4).

#### Step 4: producing program components and materials

The aim of step 4 is to develop program materials and pretest those program materials. All program materials are based on the identified methods and strategies from step 3. Here the project team consults with the healthcare professionals of the expert advisory board to determine their preferences for program design; here done via online-feedback form. The program materials are reviewed with inclusion of MBM materials, evidence-based contents and methods, books, and past courses. A pretest of the program materials supports the final production process.

#### Step 5: planning program adoption, implementation, and sustainability

A feasibility study evaluates the program’s feasibility during working hours, practicability and alignment with the healthcare professionals. Details can be found in the corresponding publication [24]. Parallel to the development of the program, efforts were also made during the entire funding period to implement it sustainably, independently of the project’s funding period:

- The management of both participating hospitals (Charité and Immanuel hospital) was involved during the entire process.
- The health insurance fund that financed this project was informed at least twice a year about the current status of the project and long-term implementation and sustainability were jointly considered.
- The other members of the various advisory boards were also involved in these processes.

#### Step 6: planning for evaluation

The evaluation plan of the feasibility study for the here developed *LAGOM* program is published elsewhere [24]. Currently, *LAGOM* is undergoing effectiveness testing [25].

## Results

### Step 1: needs assessment and program goal

**Fig. 2:**
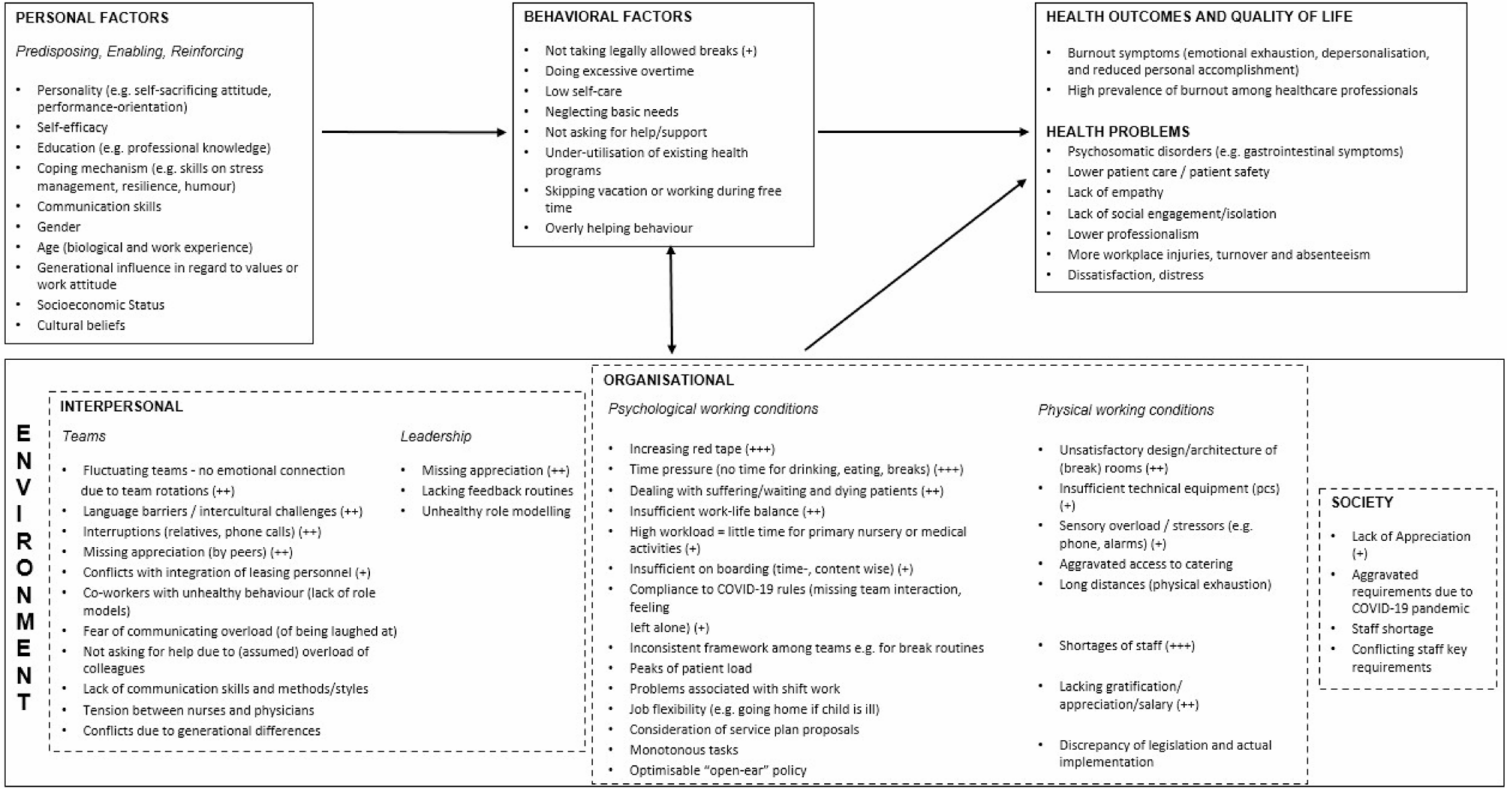
Step 1 Needs assessment, logic model of the problem as adapted from the PRECEDE model by Green & Kreuter 2005; + to +++ = weighted by frequency of mention​

Figure 2 shows the logic model of the problem. Many factors that promote burnout were identified at the behavioral level. Environmental factors also play a major role in the development of burnout: on an interpersonal, organizational and on a societal level. Derived from the needs assessment the core project team defined the following overall program goal:

*A reduction in burnout scores by the end of the LAGOM program among participants.*

### Step 2: program outcomes and objectives (logic model of change)

Tables 1, 2, 3, 4, 5, 6, 7 and 8 show the logic models of change for topic 1 to topic 8 that are based on the behavioral factors as identified in *step 1*. Based on changeability and relevance, the project team agreed on the following environmental topics (topic 9 to 13) to be included in *LAGOM*: (1) Missing appreciation by peers (team-level); (2) Lack of communication styles and methods (team-level); (3) Missing appreciation by leaders (leadership-level); (4) Lacking feedback routines (leadership-level); (5) Optimizable open ear policy (organisational-level). Those topics 9 to 13 were discussed in the steering committee and it was decided to create “stimuli” for the work environment in consultation with the board members and target group and test those within the pilot phase (step 5). Those included e.g. peer-feedback training and briefings on staff meeting (1 on 1) with focus on mental health or open-ear slots. The included environmental aspects are shown in Table 9 as *offers and impulses for your working environment*. This process was derived parallel to the IM process. At the same time the study team thoroughly elaborated external determinants (cues, reinforcement etc.) within the behavioral factors and included later methods, strategies and program components based on these learnings/prerequisites.

**Table 1:** Matrix of change objectives: performance objective 1 *Reduction of skipping breaks and taking legally prescribed break routines*

	Personal determinants					External determinants					
***Performance objectives (PO)***	Attitude (A)	Self-efficacy (SE)	Know-ledge (K)	Outcome expectations (O)	Awareness/Mindfulness (A/M)	Reinforcement (R)	Cues (C)	Stigma-tisation/Peer pressure (S/P)	Policies (P)	Autonomy (AU)	Infrastructure/Accessibility (I)
**PO.1. Reduction of skipping breaks and taking legally prescribed break routines**	**A.1** **a.** Express positive attitude towards taking breaks **b.** Trust your colleagues to have your back while you’re taking a break		**K.1** Be informed about your own rights			**R.1** Supervisors praise taking breaks			**P.1** Supervisors repeat briefings on break laws and policies (onboarding)		
**PO.1.1** Take the prescribed break time during your shift when you need it	**A.1.1** Express positive feelings towards listening to yourself and acting upon it			**O.1.1** Expect to be energized and more professional	**A/M.1.1** **a.** Notice when you need to take breaks **b.** Notice effects of taking breaks		**C.1.1** Supervisor and peers remind each other of taking breaks			**AU.1.1** Supervisors enable employees to take breaks when they need it	
**PO.1.2** Find a suitable place for a break			**K.1.2** List suitable break places								**I.1.2** Management notices the necessity of suitable break places and provides adequate resources

**Table 2:** Matrix of change objectives: performance objective 2 *Avoid the accumulation of excessive overtime*

	Personal determinants					External determinants					
***Performance objectives (PO)***	Attitude (A)	Self-efficacy (SE)	Knowledge (K)	Outcome expectations (O)	Awareness/Mindfulness (A/M)	Reinforcement (R)	Cues (C)	Stigma-tisation/Peer pressure (S/P)	Policies (P)	Autonomy (AU)	Infrastructure/Accessibility (I)
**PO.2 Avoid the accumulation of excessive overtime**			**K.2** Be informed about regulations on overtime and compensation	**OE.2** Expect positive effects of having a beneficial work-life-balance		**R.2** Supervisors reinforce ending work on time	**C.2** Supervisors regularly underline necessity to reduce overtime	**S/P.2** Peers motivate each other to keep overtime at minimum	**P.2** Management clarifies regulations clear during onboarding process		
**PO.2.1** Finish your work on time at the end of the shift	**A.2.1** Ex-press positive attitude towards finishing on time	**SE.2.1** Be confident to keep overtime to a minimum	**K.2.1** Know how to prioritize and delegate your tasks			**R.2.1** Supervisors work as role models					
**PO.2.2** Keep record of your overtime	**A.2.2** Prioritize keeping track of your working hours				**A/M.2.2** Be aware of your need for compensation	**R.2.2** Supervisors monitor overtime of the team	**C.2.2** Employees get reminded by supervisors to document their work hours.				
**PO.2.3** Communicate your overtime to your supervisor and find a timely solution to compensate		**SE.2.3** Be confident to communicate overtime to your supervisor		**OE.2.3** Expect to find a satisfying solution with your supervisor		**R.2.3** Supervisors actively communicate towards employees with overtime and offer compensation	**C.2.3** Management provides posters, brochures or email-reminders inform about compensational possibilities			**AU.2.3** Management provides a flexible way of managing overtime and their compen-sation	

**Table 3:** Matrix of change objectives: performance objective 3 *Develop and maintain self-care habits*

	Personal determinants					External determinants					
***Performance objectives (PO)***	Attitude (A)	Self-efficacy (SE)	Knowledge (K)	Outcome expectations (O)	Awareness/Mindfulness (A/M)	Reinforcement (R)	Cues (C)	Stigmatisation/Peer pressure (S/P)	Policies (P)	Autonomy (AU)	Infrastructure/Accessibility (I)
**PO.3 Develop and maintain self-care habits**	**A.3** Acknowledge your self-worth and be positive about taking care of yourself	**SE.3** Be able and express confidence to develop and maintain self-care habits	**K.3** Be informed about negative consequences of neglecting self-care	**OE.3** Expect to feel more balanced and experience the positive consequences of self-care	**A/M.3** Notice how self-care habits have an influence on your health	**R.3** Supervisors acknowledge self-care behavior and demonstrate beneficial role models	**C.3** Supervisors and Management promote self-care habits as important	**S/P.3** Peers support each other self-care strategies and avoid judging individual self-care decisions			**I/A.3** Management evaluates accessibility and optimizes the infrastructure
**PO.3.1** Take care of yourself, recognize your physical and mental needs and listen to them	**A.3.1** Prioritize taking care of yourself in situations that allow for it					**R.3.1** Supervisors evaluate self-care habits of their staff and verbalize it face to face					
**PO.3.2** Identify self-care habits that suit you	**A.3.2** Trust that your self-care habits are helpful		**K.3.2** List self-care habits you know and get to know new ones								
**PO.3.3** Make a plan for your desired self-care habits				**OE.3.3** Believe that making a plan helps to follow new routines or habits							
**PO.3.4** Establish your self-care habits			**K.3.4** Have strategies to follow habits sustainably		**A/M.3.4** Reflect which habits have the most beneficial effect						**I/A.3.4** Management provides suitable offers for self-care

**Table 4:** Matrix of change objectives: performance objective 4 *Perceiving and meeting basic needs*

	Personal determinants					External determinants					
***Performance objectives (PO)***	Attitude (A)	Self-efficacy (SE)	Knowledge (K)	Outcome expectations (O)	Awareness/Mindfulness (A/M)	Reinforcement (R)	Cues (C)	Stigmatisation/Peer pressure (S/P)	Policies (P)	Autonomy (AU)	Infrastructure/Accessibility (I)
**PO.4 Perceiving and meeting basic needs**	**A.4** Allow yourself to have basic needs	**SE.4** Express confidence to act upon your basic needs	**K.4** Be informed about professional and social consequences and health effects of neglecting basic needs	**OE.4** Expect beneficial effects for your health	**A/M.4** Take moments to perceive your current needs	**R.4** Supervisors empower their employees to act upon their basic needs					**I/A.4** Management prioritizes on improving the infrastructure for basic needs fulfillment and provides personal, social and financial resources
**PO.4.1** Recognize that satisfying your own basic needs also has a positive impact on those around you and your work				**OE.4.1** Expect beneficial effects for meeting and respecting your basic needs on your work and co-workers	**A/M.4.1** Notice the beneficial changes of you meeting basic needs for yourself and others			**S/P.4.1** Peers express appreciation for their teammates for positive changes and initiatives of change making			
**PO.4.2** Meet your basic needs						**R.4.2** Supervisors stress the importance of acting upon basic needs		**S/P.4.2** Team works together in enabling each other to act upon basic needs			

**Table 5:** Matrix of change objectives: performance objective 5 *Ask for help and support*

	Personal determinants					External determinants					
***Performance objectives (PO)***	Attitude (A)	Self-efficacy (SE)	Knowledge (K)	Outcome expectations (O)	Awareness/Mindfulness (A/M)	Reinforcement (R)	Cues (C)	Stigmatisation/Peer pressure (S/P)	Policies (P)	Autonomy (AU)	Infrastructure/Accessibility (I)
**PO.5 Ask for help and support**			**K.5** Be literate about how important asking for help is	**OE.5** Expect positive consequences by being transparent about needing help	**A/M.5** **a.** Notice your own patterns of asking or not asking for help. **b.** Reflect and accept in which areas you need help the most. **c.** Observe what changes when asking for help	**R.5** Supervisors support asking for help					**I/A.5** Management and supervisors ensure to act upon help requests
**PO.5.1** Acknowledge that it’s okay and sometimes necessary to need support	**A.5.1** Define asking for help as a sign of self-worth		**K.5.1** Know about the Caregiver syndrome								
**PO.5.2** Talk to colleagues if you need their support	**A.5.2** Be positive about team collaboration and mutual help							**S/P.5.2** Peers accept and support asking for help			

**Table 6:** Matrix of change objectives: performance objective 6 *Identification and use of existing occupational health services*

	Personal determinants					External determinants					
***Performance objectives (PO)***	Attitude (A)	Self-efficacy (SE)	Knowledge (K)	Outcome expectations (O)	Awareness/Mindfulness (A/M)	Reinforcement (R)	Cues (C)	Stigma-tisation/Peer pressure (S/P)	Policies (P)	Autonomy (AU)	Infrastructure/Accessibility (I)
**PO.6 Identification and use of existing occupational health services**	**A.6** Be open about existing offers	**SE.6** Be confident about finding an offer that works for you	**K.6** Be informed about health services that exist	**OE.6** Expect that offers have positive effects	**A/M.6** Be aware of what attracts and suits you well.	**R.6** Supervisors inform regularly on health-related offers and its importance	**C.6** Management promotes using health offers	**S/P.6** Peers appreciate it if colleagues make use of existing health services	**P.6** Management implements incentives for the use of occupational health offers	**AU.6** Management ensures structures to enable being autonomous to use health offers	**I/A.6** Management offers low-threshold, flexible, matching and easily accessible health services
**O.6.1** Ask your colleagues and supervisors about existing occupational health offers			**K.6.1** Know who in your team already uses health offers	**OE.6.1** Expect that your colleagues and supervisors will support you		**R.6.1** **a.** Supervisors empower employees to look for health offers. **b.** Colleagues reinforce each other to use health offers					
**PO.6.2** Contact the relevant health service offices			**K.6.2** Know the responsible contact details								**I/A.6.2** Management ensures that all information about health offers is updated and visible and responsible contact persons are available
**PO.6.3** Plan your desired health activities		**SE.6.3** Be confident to create a realistic and implementable plan	**K.6.3** Gain knowledge to make plans and set goals								
**PO.6.4** Talk about your plans with your supervisor and discuss possible implementations		**SE.6.4** Be confident to represent your health plans in front of your supervisor				**R.6.4** **a.** Supervisors are open to discuss plans **b.** Supervisors try to make plans work					
**PO.6.5** Take part in the offers	**A.6.5** Be committed to take part		**K.6.5** Know what works for you		**A/M.6.5** Notice changes	**R.6.5** Peers motivate each other to take part			**P.6.5** Management officially allows employees to take part within their working time		

**Table 7:** Matrix of change objectives: performance objective 7 *Take time to relax during your free time and separate work and leisure time*

	Personal determinants					External determinants					
***Performance objectives (PO)***	Attitude (A)	Self-efficacy (SE)	Knowledge (K)	Outcome expectations (O)	Awareness/Mindfulness (A/M)	Reinforcement (R)	Cues (C)	Stigmatisation/Peer pressure (S/P)	Policies (P)	Autonomy (AU)	Infrastructure/Accessibility (I)
**PO.7 Take time to relax during your free time and separate work and leisure time**	**A.7** Express positive attitude towards taking time to relax	**SE.7** Be confident about separating work and leisure time	**K.7** **a.** Know about your rights **b.** Know different kinds of relaxation routines	**OE.7** Expect beneficial effects of recovery	**A/M.7** Notice how recovery improves your health and work situation	**R.7** Supervisors reinforce others to take time to relax (also by role modeling)	**C.7** Management reminds to take vacation and leisure time (with attached laws)	**S/P.7** Colleagues support leisure time behavior in co-workers		**AU.7** Supervisors allow autonomous team vacation determination when possible	
**PO.7.1** Turn off work-related communication during your free time and create an out-of-office notification in your e-mail account			**K.7.1** Know about the positive impact of no interruptions of your recovery time		**A/M.7.1** Notice differences when keeping your communication devices turned off	**R.7.1** **a.** Supervisors claim that employees are not available **b.** Supervisors do not contact employees during their free time **c.** Supervisors as well as employees refrain from work during their free time		**S/P.7.1** Colleagues respect times of non-availability			
**PO.7.2** Plan activities that will allow you to mentally disconnect	**A.7.2** Take your free time activities seriously	**SE.7.2** Be confident to adhere to your plans			**A/M.7.2** Reflect which activities help you to disconnect						
**PO.7.3** Create rituals at the end of your workday	**A.7.3** Be positive about creating rituals to help you resist patterns that keep you working			**OE.7.3** Expect that it’s easier to end your shift	**A/M.7.3** Notice the patterns that keep you working after your shift end						

**Table 8:** Matrix of change objectives: performance objective 8 *Communicate personal capacities among colleagues and act in alignment with them*

	Personal determinants					External determinants					
***Performance objectives (PO)***	Attitude (A)	Self-efficacy (SE)	Knowledge (K)	Outcome expectations (O)	Awareness/Mindfulness (A/M)	Reinforcement (R)	Cues (C)	Stigmatisation/Peer pressure (S/P)	Policies (P)	Autonomy (AU)	Infrastructure/Accessibility (I)
**PO.8 Communicate personal capacities among colleagues and act in alignment with them**	**A.8** Be positive about communicating your personal capacities	**SE.8** Be self-confident about communicating your capacities	**K.8** Know communication skills to express your capacities			**R.8** Supervisors empower employees to communicate their personal capacities		**S/P.8** Peers support colleagues in communicating capacities			
**PO.8.1** Identify your personal resources		**SE.8.1** Trust yourself in identifying your personal resources	**K.8.1** Know how to identify your personal resources and be aware that they are individual and fluctuate			**R.8.1** Supervisors stress the importance of personal resources and limitations and provide opportunities to reflect on it		**S/P.8.1** Peers respect their colleagues’ personal resources and limitations			
**PO.8.2** Acknowledge your limitations and take them seriously			**K.8.2** Be informed about positive consequences of taking limitations seriously	**OE.8.2** Expect an improvement in your work environment	**A/M.8.2** Reflect upon your limitations from time to time						
**PO.8.4** Check if your help is needed and accept that there may be situations where others can handle the situation on their own					**A/M.8.4** Be aware of your own expectations and check them against reality						
**PO.8.5** Learn to say “no”	**A.8.5** Have a positive attitude towards saying no	**SE.8.5** Be confident about saying no						**S/P.8.5** Peers accept colleagues saying no			

**Table 9:** Linking of methods, strategies, and program component with change objectives

Change objectives	Method	Strategy	Program Component
Behavior			
**A.1** **OE.2.3** **A.3** **OE.5** **A.7** **K.7b** **A/M.7**	Shifting perspective	Documentation of thoughts Focusing on positive experiences Reframing Cognitive Restructuring	Enjoyment training (e.g. raisin exercise) ABCD scheme (examples of everyday clinical practice) Transformation of beliefs (Reframing: I always have to, I can always, I can sometimes, I may) Resource shower (self-confidence e.g. self-esteem bouquet, appreciative feedback)) Leisure resources list (e.g. list things that are good for me and put them on the fridge) Work End Ritual (positive) Observation task e.g. “pay attention next week to what succeeds/does well/goes according to your wishes”. (e.g. experiencing asking for help) Circle of influence on flipchart/graphics (acceptance training) “What can you change, do you want to. What not” STOP-Method – Interruption Mechanism/Stress Automatisms (Mindfulness) Stop-Breath-Think-Precede
**A.1.1** **A/M.1.1** **A.2.1** **A/M.2.2** **A/M.3.4** **OE.1.1** **K.2.1** **A.5.1/A5.2** **A/M.6.5** **A/M.7.3** **A.7.3** **A.8** **A/M.8.2/A/M.8.3** **R.2.2** **C.2/C.2.2**	Self-evaluation, Self-reflection	Self-test/-awareness Reflection rounds, scales Documentation Exchange in the group	Self-awareness (physically), self-experience Diary (documentation of breaks, burnout signs, reflection on which exercises fit, physical effects, energy diary, happiness diary), Journaling Inner Team-Work Stress Warning Signals Test Stress-Intensifying Thought Questionnaire/Reframing Reflection on Life Net (with social contacts, free time, fulfillment of meaning, job, money) Electrophysiological measurement or with wearables Check in/Check out (before, after course) – “Mental check in” as a method (for the team) Inner team constellation (make inner parts clear, helpful, perfectionist etc.)
**K.8**	Persuasive communication	Credible, comprehensible information in e.g. script/workbook, slide sets/presentations/Sources, Graphics	Script/workbook for participants or slide sets, handout with information Communication techniques/rhetoric, negotiation (external posture, voice, facial expressions/gestures for more confidence, high status, low status, power poses)
**K.3.2**,** K.3.4** **K.1.2** **K.2.1** **SE.8.1** **K.8.1**	Active processing of information	Self-development of topics, active/independent elaboration, practice	Stress/burnout facts and early warning signs (handout, slides) Slides with background information with sources, individualized “Resource treasure box” 4 Ears Communication Model – test on communication-type
**A.6**	Repeated exposure	Information (available, accessible)	Information on existing health services in the script/handout/flyer Newsletter Screensaver
**SE.2.3** **K.5** **OE.5** **A/M.5** **SE.6** **K.6.5** **A/M7.1** **A/M7.2** **A.8/A.8.5** **SE.8/SE 8.5** **OE.8.2**	Direct experience	Exchange in the group and reinforcement self-experience Testing of content in everyday life, exercises for everyday life Pursuit of own goals	Concept of salutogenesis, coherence Games e.g. Team Building –guiding a “blindfolded” team member Observation task/trying out new strategies in everyday life and sharing experience (e.g. asking for help) Take advantage of sneak offers (courses Charité or Immanuel) & evaluate them “Cell phone-off-evening” and gain experience Gong app for self-reflection moments during the day Partner exercise: Saying no to absurd questions/humorous Try it out in your circle of friends/partners or with your buddy, to say “no” more often
**A.3.2** **OE.3** **SE.4** **K.5.1** **OE.6.1**	Modelling	Creating role models, (trainers/managers) (communication, etc.), demonstrating exercises/impulses	Peer exchange in the course on effects Interaction with course trainer Briefing and supervision of trainer Peer exchange on self-care routines (insight dialogue) Humorous sequence on basic needs “Joint lunch break”/agreement of managers – healthy lunch culture
**A.3.1** **OE.3** **OE.2** **A/M.3** **OE.1.1** **K.7.1**	Guided practice	Live exercises, feedback, role plays, and relaxation techniques	Metta Meditation (Self-Compassion) Movement impulses for everyday life/break (active break, suitable exercises for nurses/doctors, coordination exercises) Breathing Minis Exercises in the course Recipes for healthy meals and bring-your-own buffet in the course Showing naturopathic self-care routines (e.g. with essential oils) in the course Relaxation methods (e.g. PMR, or other meditations) Letter or inspirational note to oneself/benevolent letter in self-kindness (recommendations to oneself as well as to best friend) Self-massage (ears, hands, feet, relaxation points)
**Se.2.1** **K3.4** **Oa.3.3** **SE.3** **A.2.2** **A/M.4.1** **A.6.5** **SE.6.3** **K.6.3** **A.7.2** **SE.7.2** **OE.7.3**	Self-regulation	Goal setting	AROMA goals or healthy routines/rituals Goal adjustment (evaluation of importance, confidence – influence, ease of implementation – clarify the WHY, what is the purpose of this, Inhibition management/relapse prevention) - Scaling based on HAPA model and transtheoretical model Planning a “happiness health project” Reflection questionnaire – what has changed after time X, evaluating expectations and actual experiences Celebrating successes and planning rewards
**A/M.4** **SE 6.4** **K.7b**	Planning coping responses	Stress coping models, interruption techniques (distancing)	Small micro-exercise to feel physical and psychological needs (consciousness for own breath) Mindful walk to work or between rooms Body-related meditation Regular gong reminders to check in inside yourself (what do I need right now?) - suitable for everyday hospital life Own Health/health plan with plans (courses Charité/Immanuel etc.) for optional discussion with superiors
**OE.2** **K.3** **K.4** **A.4** **OE.4/OE.4.1** **OE.6** **K.8.2**	Arguments/information/tailoring	Psychoeducation	Information on stress response to feelings, thoughts, etc. stress regulation/relaxation response, e.g. Karasek model (job demand/control) Effect of chronic stress/impact of non-fulfillment of needs (body effects) - Positive effects (self-care) for Individual and team – examples in everyday clinical practice Need/emotional vocabulary - Addressing basic needs (psychological) in relation to burnout/stress
**A/M.3.4** **A.4** **K.6.1** **SE.7** **OE.7** **A/M.7** **A/M.8.4**	Discussion	Interaction in group (e.g. moderated)	Exchange after relaxation exercises (effect), about recovery strategies/experiences Reflection rounds in the course on experiences Practicing active listening/empathic listening Exchange on the use of health services so far Scaling/running in room
**External**			
**K.6** **K7.a** **P.1** **R.2** **R.2.2** **C.2.3** **P.2** **AU.2.3** **R.3** **C.3** **I/A.3** **R.4** **C.6** **P.6.5** **AU.6** **I/A.6** **I/A.6.4** **R.7.1** **C.7** **S/P.7 + C/P 7.1** **S/P 8.5**	Cues	Posters, calendars, reminders at work and in the course	Desk calendars, information leaflets with occupational health and safety rules, overtime regulations or newsletters, brochures if necessary Screensavers with information/information-mails from Human Resources (e.g. for breaks, holidays, overtime compensation/regulations) + documentation systems (CHEP, PEP) Creative messages e.g. “Everyone has a psyche” - “LAGOM-ed today?” on the topic “Self-care is important”, “Saying no is a yes to…” Recipes with self-care-taking, quick recipes for working professionals and shift work Read/inform yourself about LAGOM newsletter on health offers + corresponding contact persons + independent regulation (autonomous, self-employed) (rules working time = course time, vacation minutes) Poster with LAGOM basics for wards (selfcare, breaks, after-work, holidays) Postcard with Memo LAGOM 5 Mental Health Tips for Employees (Cards for managers and employees) Email signature Onboarding videos (inspirational videos) LAGOM-appropriate further training offers for specialization as a flyer/information sheet Massages Sitting time interrupter (smart watches, smartphones) Merch/branding
**A.3** **R.7.1**	Non-judgmental group discussions	Group rules Feedback Conflict management	Appreciative group rules, rules for feedback Discussing critical subjects (taking breaks, taking leisure time seriously, accessibility) - discussion format
**K.2.1** **OE.6.1** **S/P.3** **S/P.4.1** **S/P.4.2** **I/A.4** **S/P.5.2** **R.6.1b** **R.7.1** **S/P.8/8.1**	Stimulate communication and mobilizing social support	Exchange groups (interprofessional) Messenger groups, chat, support networks (for duty rosters, outage etc.) Mental Health Support Groups/Mental Health positive community	Draw the social network (make the team’s competencies clear) or the support network map Buddy system within the program - motivate colleagues to use self-care offers Self-coordinated exchange group (course) - support system with like-minded people – LAGOM-“Family”/Group (share ideas, inspire each other, motivate each other) Peer -feedback concept (2–3 peers from your own team who do not participate give feedback within the time 2–3 times to improve self-care) Long-term LAGOM/Mental Health Support Group with LAGOM mentors/ambassadors
**R.1** **C.1.1** **AU.1.1** **R.2/R.2.1** **R.3** **R.4.2** **R.5** **R.6.4.a + b** **R.7**	Model availability	Team Ambassadors Buddy Systems Peer Groups Video Models Minister of Health (Theory U)	“Happy Hour” once a month, e.g. 1–2 h lunch break together with your ward Setting an example of healthy break behavior - authenticity - sense of responsibility - discussion/example if necessary how bad is it when managers do not take a break Letter of Intent/Letter of LAGOM for each participant with LAGOM – Basics, which is signed by superiors (LAGOM basics such as working hours/training, asking for help, basic needs, break, being a role model yourself, support of plans) - - Information sheet “what does LAGOM mean - participation with your employee”
**S/P.4.1**	Advocacy	Press conference, information events, mentoring programs Participatory problem solving (Open Ear Policy)	Health ambassadors, LAGOM coaches (long-term) Information and workshops on course for leadership/superiors Open Ear Policy from decision makers (see below)
**I/A.5**	Participatory problem solving		Open Ear Policy – visible/accessible contact persons
**I/A.1.2**	Effective change management	Chief Happiness Officer Vision board on employee level	Creating own vision board e.g.: what does a good working atmosphere look like, such as a more mentally healthy/self-care station
**P.1** **R.2.3**,** R.3.1**,** R.4.2**,** R.6**,** R.6.a** **R.8.1**	Skills training	Mental health training Communication training and workshops	Employee 1on1s including mental health, mental health checkup (discuss resources), break behavior or overtime behavior, e.g. during onboarding) - if necessary, sensitize in own team Conversational skills/feedback/communication techniques, empathy, active listening
**S/P.2** **R.6.1a** **R.8**	Persuasive Communication	Conducting interviews (outsourcing) Boundary setting	Quick-wittedness, developing autonomous skills, making humorous/strong response strategies/skills for self-confidence during breaks (vs. peers), Healthy boundaries – who is good for me, who is not Setting inner boundaries - give feedback, e.g. to managers about break behavior or ask about how they are feeling Leadership training: Empowering employees for mentally healthy behavior
**R.6.1b** **R.6.5** **S/P.6** **P.6** **P.6.1** **P.6.5**	Incentives	Coffee, tea, working hours, break room, vouchers, fruit, water dispenser	Paid course e.g. yoga Course time is counted as working time Bring your colleague - Incentives for self-care training - Bring a colleague with you and get a coffee voucher/or massage Gamification– with this course you get e.g. hoodie, merch, coffee voucher
**I/A.1.2** **I/A.3.4** **I/A.4** **R.7.1** **Au.7**	Rooms/Architecture	Rooms with equipment Personnel infrastructure	Financial budget for feel-good atmosphere or for break room per year (water dispenser, coffee machine, etc.) LAGOM tips (flyer) for break room improvement (catering, water dispenser, light) − 10 points check list New position e.g. LAGOM Chief Happiness Officer contact person Technical setup – protection of free time (no email access, own work number, etc.) Technical system for recording overtime

### Step 3: program design

Table 10 shows the linking of methods and strategies with each identified change objective from step 2.

**Table 10:** Schedule of the 9-week *LAGOM* program revised after feasibility testing

Session	1	2	3	4	5	6	7	8	9
Time	180 min	90 min	90 min	90 min	90 min	90 min	90 min	90 min	180 min
20 min	Activating Movement – “Minute for Me” - Reflection								
Topic	**Introduction to the LAGOM program – “so that work is fun"**	**“Yes! But…” -** **Behavior Change and Goal Setting Made Easy**	**“Take breaks like a pro” - healthy routines in everyday life**	**“Don’t believe everything you think…” - Power of Thoughts**	**“Who am I and if so**,** how many?” - managing & developing the inner team**	**“I hear something you don’t say” - communication with others**	**“I’m also there for myself” - a lived balance between self-care and caring for others**	**“Future Values in the World of Work” - Rethinking & Feeling Organization**	**“The end of the course is the beginning of…?” - Outlook**
Format	*(Presence)*	*(Online)*	*(Presence)*	*(Online)*	*(Presence)*	*(Online)*	*(Presence)*	*(Online)*	*(Presence)*
50 min	Introduction, getting to know each other Temple of Health Burnout/Resilience	Stages of Behavior Change AROMA Target Formulation (Health Goal)	Stress and stress patterns Breaks and active self-care	Cognitive Regulation: Power of Thought ABCD Scheme	Identifying Inner shares Recognizing inhibiting/promoting parts and intentions	Empathetic, conscious listening Feeling Finder & Needs Compass 4-Ear Model for Stressor Detection	Protection and mindful use of one’s own resources Self-Care & Self-compassion	Work and organizational culture Visionboard, reflection on your own work	Graduation, Celebrating Successes, Summary Planning a happiness project/health project Follow Up: Buddy Group
15 min	Relaxation or movement exercise for everyday life								
	Progressive Muscle Relaxation	(Breathing-) Minis	A place of peace and strength	Meditation on Thoughts	Breathing-meditation	Enjoying/meditative walking	Say „No“ Exercise	Meditation on Feelings	Compassion Meditation
	Breathing and walking exercise	Energizing through acupressure	Naturopathic self-care strategies	Eye Yoga	Yoga for the break, Exercises in the workplace	Stretching/stretching exercises while walking	Powerposes	Self-massage	Conscious dialogue (Buddies)
	Deepening at home								
5 min	Stress warning signs, Energy diary Desk calendar, Study folder	Stress-exacerbating thought questionnaire	Try recipes/routines	Observation according to ABCD Scheme	Free Writing/Journaling Setting up the inner team	Communication test	Creative anchoring	Visionboard	Letter to Yourself Conclusion, Follow Up
Recipes	*Energy balls/healthy snacks*	*Power spreads*	*10 Nutritional* *tips & Quick break salads*	*Start of the day*	*Smoothies & Drinks*	*Heartwarmer soups*	*The Sweet Life*	*For the big superhero: inside-hunger*	*Bring in your own recipes*
	**LAGOM - Offers and impulses for your working environment**								
	1	2	3	4	5	6	7	8	9
	Information on company offers	Peer-Feedback Concept	Tips for a healthy break/improving the break (room)	Mental health check-up appointment (employee interview - guidelines)	External massage offer	Conflict resolution services for teams (external mediation) Offers collegial case counseling/team coaching/supervision	Healthy snack box options for wards	Tips for better meetings	“Happy Hour” at your own station - interprofessional lunch break

### Step 4: producing program components and materials

**Fig. 3:**
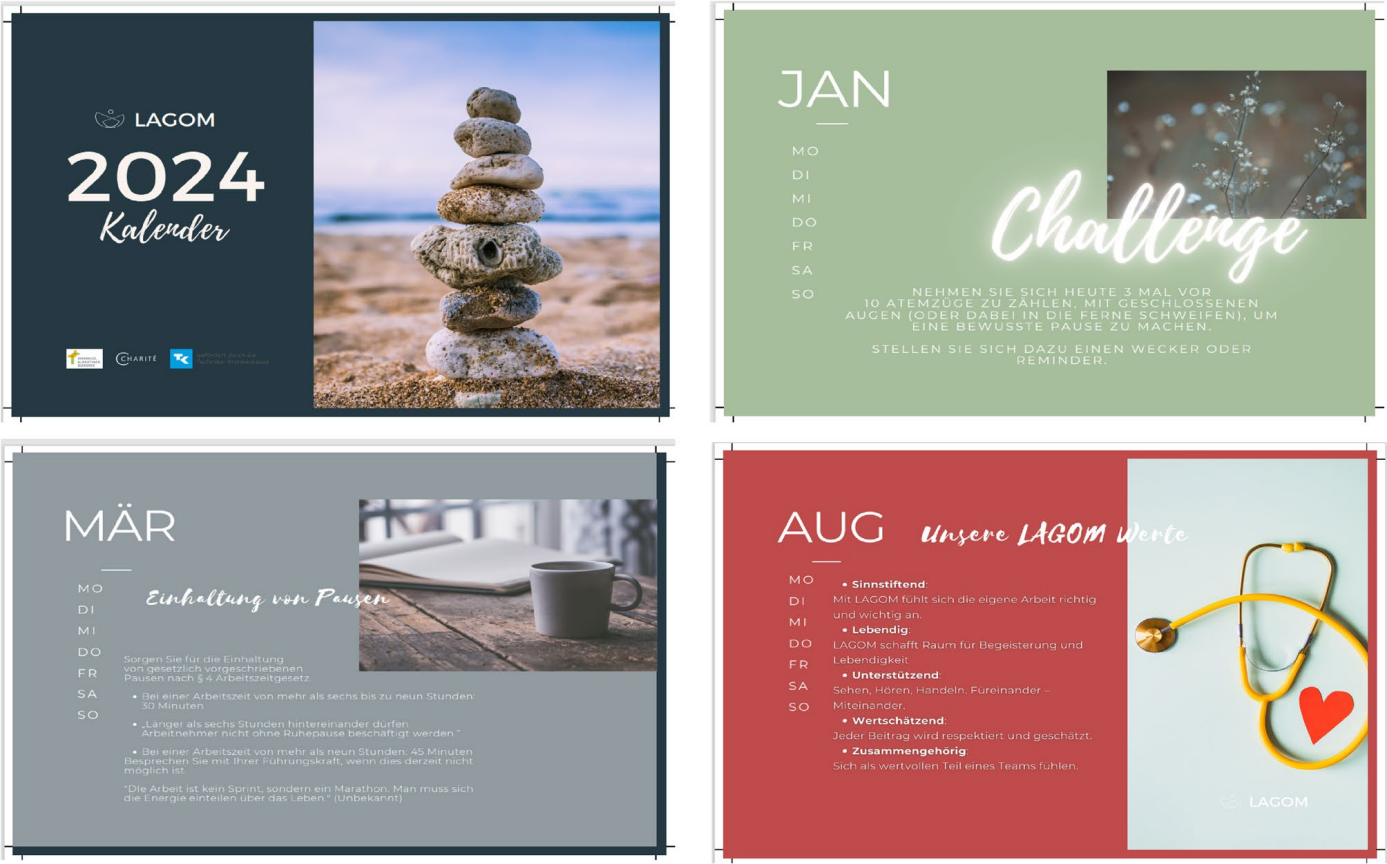
*LAGOM* desk calendar (cutout)

**Fig. 4:**
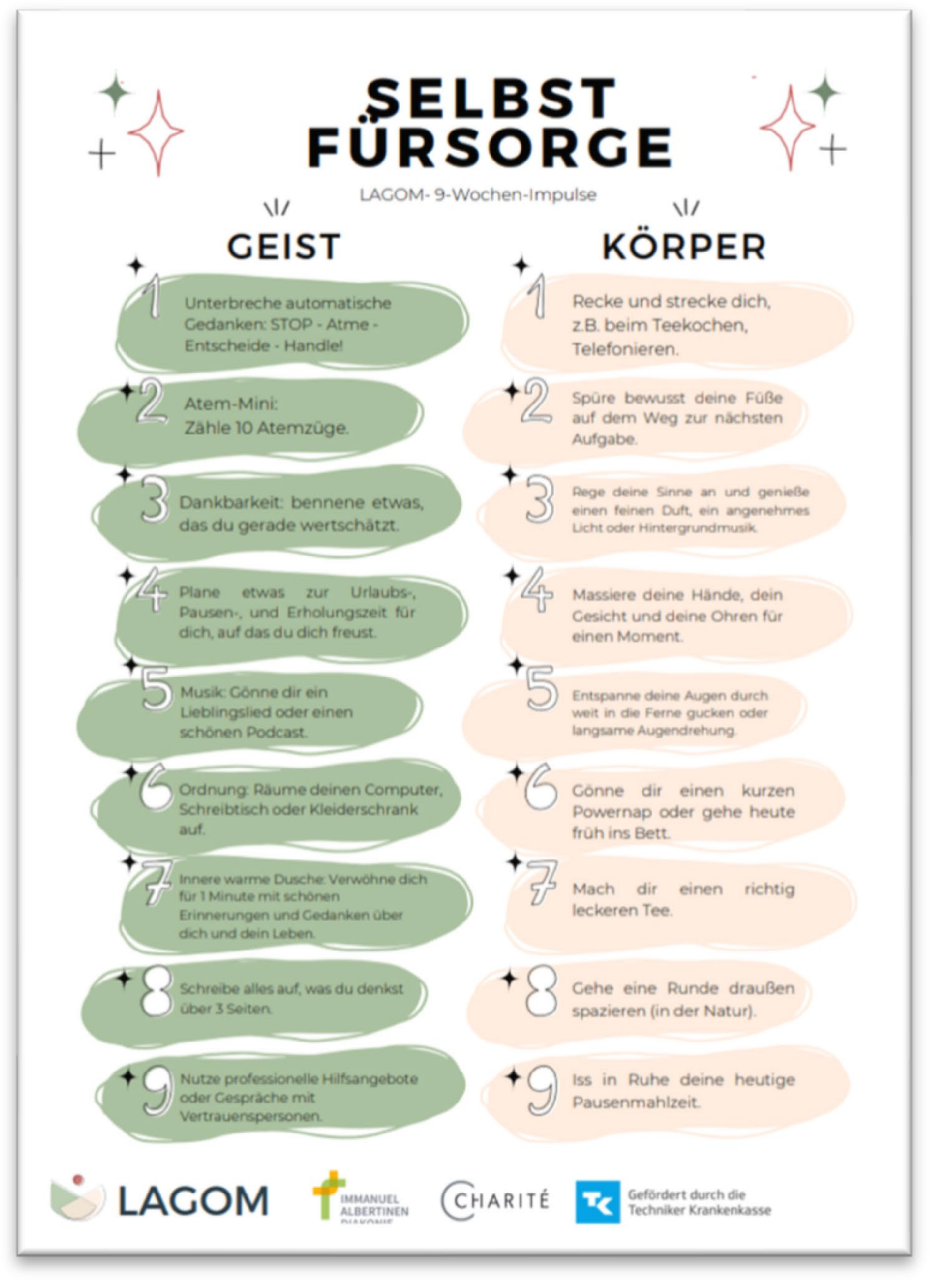
*LAGOM* poster

The final *LAGOM* program consist of nine sessions that include structural and behavioral prevention. Table 9 shows the schedule of the 9-week *LAGOM* program revised after feasibility testing [24]. Each session will follow the same structure: [1] psychoeducational and interactive part on different topics with practical exercises and group exchange [2], a relaxation or movement exercise [3], session conclusion and invitations for individual deepening of subjects and a recipe of the week. The individual training components are accompanied by work field impulses. This takes place either directly on site during the sessions or externally, for example through emails. The weekly *LAGOM* sessions are led by qualified and experienced trainers with MBM background and specially trained for the course adaption. Figure 3 shows excerpts from the *LAGOM* desk calendar, Fig. 4 shows the *LAGOM* poster. More details on the intervention content can be found at Schröter, Berschick [24] and Koch, Schröter [25].

### Step 5: planning program adoption, implementation, and sustainability

Various steps have been undertaken to ensure the sustainable implementation and dissemination of *LAGOM*. A key prerequisite for workplace prevention is management-level support by the steering committee, which was secured in the *LAGOM* project through active involvement and support from top management. Workshops were held at the Charité and the Immanuel Hospital to introduce the program and the associated evaluations to the managers of the participating institutions. Collaboration with sustainability management and change management was established, and the *LAGOM* homepage was linked to the sustainability management website to ensure its continuity beyond project funding. Focus groups and expert advisory board as well as the steering committee were convened to gather insights, supervision and feedback during the content creation period. Interdisciplinarity was fostered to enhance the project’s robustness and relevance across various organizational functions and medical fields. Feasibility assessments were conducted to refine *LAGOM* [24], ensuring its practicality and effectiveness in real-world settings.

### Step 6: planning for evaluation

The detailed evaluation plans for the feasibility study as well as for the effectiveness study are published elsewhere [24, 25].

## Discussion

The *LAGOM* program aims to prevent burnout among healthcare professionals in hospitals by means of behavioral and structural prevention. The program was developed with the help of the addressees in a structured manner based on the six steps of the IM. This paper illustrates in detail the preparatory work and the individual steps of the IM in combination with the PRECEDE-PROCEED logic model that led to the development, testing and implementation of the *LAGOM* program. The program was co-developed with an expert advisory board including healthcare professionals and a steering committee with participants on executive level as well as tailored to the specific realities of two hospital settings. It addresses the critical gap, as shown in a systematic review, of limited availability of evidence-based interventions that explicitly target organizational and system level factors [28]. This is of particular importance as one of the main causes of burnout is poor working conditions [29], which is also reflected in the results of our needs assessment. Numerous methodological approaches have been developed to guide the design and evaluation process of interventions [30, 31]. Multiple ways of theorizing interventions have been suggested, such as RE-AIM model or Behavior Change Wheel, as well as theory-driven evaluations [32–36]. Grounding interventions in a theoretical framework has been strongly suggested as it enables the formulation of a program theory explaining how and why an intervention is expected to produce its intended outcomes [30, 37]. While Behavior Change Wheel and RE-AIM model focus more on either design or evaluation, IM provides a full-process guidance. Besides IM, one of the most widely adopted frameworks for theory-driven, full-process guidance interventions in nursing and healthcare research is the Medical Research Council (MRC) Framework. IM and MRC both have strengths and weaknesses and the MRC framework has been updated in recent years to address critiques, e.g. regarding contextual sensitivity [37]. While IM doesn’t allow for as much flexibility in theoretical guidance and the systematic development steps as MRC, it offers a highly structured, detailed and prescriptive development process. Despite potential lower flexibility, this rigor was considered as a strength in our context as it supports a theoretically coherent and contextually grounded process. To further enhance our chosen approach, we included the PRECEDE-PROCEED model as it extends understanding of what needs to be changed and why, which is then translated through the steps of IM by operationalizing diagnostic insights into actionable intervention content. Especially for healthcare interventions which are widely recognized as complex the combination of IM and PRECEDE-PROCEED Logic model ensures a robust implementation and evaluation strategy and has been suggested [21, 30, 36]. A notable strength of the *LAGOM* program is its participatory design. Collaboration between the addressees of the intervention, the management level and the project team is crucial for successful development and implementation. Engaging stakeholders from all levels not only ensures that the intervention is contextualized, but also fosters a sense of ownership and collective responsibility for burnout prevention within an organization. Iterative project design and adaptation provides the opportunity to refine interventions based on ongoing feedback and evolving needs. Continuous feedback and flexibility are also important to maintain relevance of the intervention over time, especially in a dynamic and high-pressure environment like hospital settings. This participatory approach aligns with findings that interventions co-developed with the population they are intended for are more likely to succeed due to higher relevance and perceived control [8]. Furthermore, the applied approach ensures transparency for various stakeholders such as health professionals, researchers, practitioners and policy makers who wish to understand, evaluate or replicate the program.

This study also has some limitations. As important as the integration of behavioral and structural prevention is: Effective structural prevention in hospitals requires the involvement of many different stakeholders and the acquisition of mostly non-existent financial resources. E.g. as the involvement of staff placed additional demands, participation of the expert advisory board fluctuated considerably despite welcoming opportunities to articulate their workplace challenges and needs. In the LAGOM project, many people from the management level worked on the steering committee, supporting the project and trying to make many things possible. For example, it was ensured that all interested employees were able to participate in the program during their working hours. However, within the needs analysis it becomes clear that there is a greater need to improve structures than can be covered by the resources of this program. Personnel shortage, increasing red tape as well as severe time constraints, which often prevent staff from taking adequate breaks, including time to eat or hydrate, reflect systemic challenges that necessitate not only organizational level changes but also broader structural and policy reforms. The *LAGOM* team had to focus on certain aspects in order to ensure feasibility. This, while important for immediate intervention, also highlights the challenge of addressing the full scope of burnout’s root causes in terms of available resources. However, the structures created by the project and the knowledge generated about the prevailing problems in the two hospitals will enable these additional topics to be addressed in the future.

Structured checklists for reporting the structured development of complex interventions would have great added value. Such checklists would improve transparency and replicability and furthermore allow other institutions to adapt and scale the intervention more effectively, leading to broader, system-wide improvements in healthcare professionals´ well-being.

The findings from this study underline the need for continued research on integrated burnout prevention strategies. IM guides the design of interventions at the behavioral and structural prevention levels by identifying barriers, selecting intervention components, applying theories, and effectively engaging end users. IM requires a significant amount of time, expertise and stakeholder engagement which poses challenges to implement this complex process in fast-paced healthcare environments. This is particularly critical when there is a notable gap between the resources allocated for intervention development and the practical feasibility of implementation due to constrains by structural or organizational limitations. This gap highlights the need for further research and practical applications focusing on structural interventions for healthcare professionals which have the potential to address burnout at a larger, systemic scale. Furthermore, future studies should explore how interventions like *LAGOM* can be adapted for different healthcare professions and contexts and evaluate long-term outcomes, including organizational metrics such as staff retention, patient satisfaction, and overall workplace well-being. The success of structural components may take longer to manifest and require sustained institutional commitment.

Policy implications should not be overlooked. Institutional support and policies promoting mental health and well-being must be prioritized to enable the successful implementation and sustainability of such interventions. That also includes addressing systemic barriers such as establishing adequate staffing levels. Further recommendations include the integration of co-design approaches, as they have been a valuable source to ensure success as well as establishing reporting standards that include structured checklists for transparency.

## Conclusions

The IM framework proved effective in systematically addressing the complexity of burnout prevention. Its application in other healthcare settings could help develop interventions that balance individual and structural components, tailored to the unique needs of each organization.

The *LAGOM* program demonstrates the potential of a comprehensive, participatory approach to burnout prevention in the healthcare setting. By addressing both individual and structural factors, it offers a promising model for creating healthier work environments. However, its effectiveness depends on sustained engagement from both employees and leadership, highlighting the importance of a supportive organizational culture. Future research should focus on long-term implementation strategies and broader application across diverse healthcare professions and settings to further advance burnout prevention efforts.

The following key messages outline the central implications. Both individual and structural conditions should be targeted to enable effective burnout prevention. Co-developing interventions is essential to ensure contextual sensitivity and relevance. Institutional and policy support has shown to build a solid foundation for implementation and sustainability. Future research should focus on long-term outcomes while the development of robust implementation strategies is essential to ensure effective translation into everyday practice.

## Supplementary Information

Supplementary Material 1

## Data Availability

The datasets generated and analysed during the current study are not publicly available to protect study participant privacy but are available from the corresponding author on reasonable request.

## References

[1] Hodkinson A, Zhou, Anli, Johnson J, Geraghty K, Riley R, Zhou A, et al. Associations of physician burnout with career engagement and quality of patient care: systematic review and meta-analysis. BMJ. 2022;378:e070442. 36104064 10.1136/bmj-2022-070442 PMC9472104

[2] Fahrenkopf AM, Sectish TC, Barger LK, Sharek PJ, Lewin D, Chiang VW, et al. Rates of medication errors among depressed and burnt out residents: prospective cohort study. BMJ. 2008;336(7642):488–91. 18258931 10.1136/bmj.39469.763218.BE PMC2258399

[3] Shanafelt TD, Balch CM, Bechamps G, Russell T, Dyrbye L, Satele D, et al. Burnout and medical errors among American surgeons. Ann Surg. 2010;251(6):995–1000. 19934755 10.1097/SLA.0b013e3181bfdab3

[4] West CP, Tan AD, Habermann TM, Sloan JA, Shanafelt TD. Association of resident fatigue and distress with perceived medical errors. JAMA. 2009;302(12):1294–300. 19773564 10.1001/jama.2009.1389

[5] Shirey MR. Stress and burnout in nursing faculty. Nurse Educ. 2006;31(3):95–7. 16708030 10.1097/00006223-200605000-00002

[6] West CP, Shanafelt TD. Physician well-being and professionalism. Minn Med. 2007;90(8):44–6. 17899849

[7] Dyrbye LN, Massie FS, Eacker A, Harper W, Power D, Durning SJ, et al. Relationship between burnout and professional conduct and attitudes among US medical students. JAMA. 2010;304(11):1173–80. 20841530 10.1001/jama.2010.1318

[8] Shanafelt TD, Mungo M, Schmitgen J, Storz KA, Reeves D, Hayes SN, et al. Longitudinal study evaluating the association between physician burnout and changes in professional work effort. Mayo Clin Proc. 2016;91(4):422–31. 27046522 10.1016/j.mayocp.2016.02.001

[9] Dyrbye LN, Shanafelt TD. Physician burnout: a potential threat to successful health care reform. JAMA. 2011;305(19):2009–10. 21586718 10.1001/jama.2011.652

[10] Velana M, Rinkenauer G. Individual-level interventions for decreasing job-related stress and enhancing coping strategies among nurses: a systematic review. Front Psychol. 2021;12:708696. 34349711 10.3389/fpsyg.2021.708696 PMC8326445

[11] West CP, Dyrbye LN, Erwin PJ, Shanafelt TD. Interventions to prevent and reduce physician burnout: a systematic review and meta-analysis. Lancet. 2016;388(10057):2272–81. 27692469 10.1016/S0140-6736(16)31279-X

[12] Schiele JK, Koch AK, Adam D, Berschick J, Schröter M, Reschke S, et al. Vom burnout Zur balance. Programme in Deutschen krankenhäusern: Graue literaturübersicht Mit semi-strukturierten interviews (from burnout to balance. Programs in German hospitals: grey literature review with semi-structured interviews). Z Arbeitsmed Sozialmed Umweltmed. 2023;59:38–45.

[13] Zhang X-j, Song Y, Jiang T, Ding N, Shi T-y. Interventions to reduce burnout of physicians and nurses: an overview of systematic reviews and meta-analyses. Medicine. 2020;99(26):e20992. 32590814 10.1097/MD.0000000000020992 PMC7328917

[14] Pijpker R, Vaandrager L, Veen EJ, Koelen MA. Combined interventions to reduce burnout complaints and promote return to work: a systematic review of effectiveness and mediators of change. Int J Environ Res Public Health. 2020;17(1):55. 10.3390/ijerph17010055 PMC6981402 31861699

[15] Dreison KC, Luther L, Bonfils KA, Sliter MT, McGrew JH, Salyers MP. Job burnout in mental health providers: a meta-analysis of 35 years of intervention research. J Occup Health Psychol. 2018;23(1):18. 27643608 10.1037/ocp0000047

[16] Cohen C, Pignata S, Bezak E, Tie M, Childs J. Workplace interventions to improve well-being and reduce burnout for nurses, physicians and allied healthcare professionals: a systematic review. BMJ Open. 2023;13(6):e071203. 10.1136/bmjopen-2022-071203 PMC10314589 37385740

[17] Maresca G, Corallo F, Catanese G, Formica C, Lo Buono V. Coping strategies of healthcare professionals with burnout syndrome: a systematic review. Medicina (B Aires). 2022;58(2):327. 10.3390/medicina58020327 PMC8877512 35208650

[18] Awa WL, Plaumann M, Walter U. Burnout prevention: a review of intervention programs. Patient Educ Couns. 2010;78(2):184–90. 19467822 10.1016/j.pec.2009.04.008

[19] Bes I, Shoman Y, Al-Gobari M, Rousson V, Guseva Canu I. Organizational interventions and occupational burnout: a meta-analysis with focus on exhaustion. Int Arch Occup Environ Health. 2023;96(9):1211–23. 37758838 10.1007/s00420-023-02009-z PMC10560169

[20] Ramos S, Costa P, Passos AM, Silva SA, Sacadura-Leite E. Intervening on burnout in complex organizations–the incomplete process of an action research in the hospital. Front Psychol. 2020;11:2203. 33071844 10.3389/fpsyg.2020.02203 PMC7538901

[21] Eldredge BLK, Markham CM, Ruiter RAC, Fernàndez ME, Kok G, Parcel GS. Planning health promotion programs: an intervention mapping approach. Hoboken, NJ: Wiley; 2016.

[22] Kok G, Gottlieb NH, Peters G-JY, Mullen PD, Parcel GS, Ruiter RAC, et al. A taxonomy of behaviour change methods: an intervention mapping approach. Health Psychol Rev. 2016;10(3):297–312. 26262912 10.1080/17437199.2015.1077155 PMC4975080

[23] Green LW, Kreuter MW. Health program planning: an educational and ecological approach. New York: McGraw-Hill; 2005.

[24] Schröter M, Berschick J, Koch AK, Schiele JK, Bogdanski M, Steinmetz M, et al. Feasibility of a custom-tailored, evidence-based, theory-informed, intervention to prevent burnout and reduce stress for healthcare professionals: protocol for a single-arm trial. Pilot Feasibility Stud. 2024;10(1):134. 39511690 10.1186/s40814-024-01553-w PMC11542239

[25] Koch AK, Schröter M, Berschick J, Schiele JK, Bogdanski M, Steinmetz M, et al. A custom tailored, evidence-based, theory-informed intervention for healthcare professionals to prevent burnout (LAGOM): study protocol for a pragmatic randomized controlled trial. Trials. 2024;25(1):628. 39334393 10.1186/s13063-024-08491-1 PMC11429380

[26] Kok G, Gottlieb NH, Peters G-JY, Mullen PD, Parcel GS, Ruiter RA, et al. A taxonomy of behaviour change methods: an intervention mapping approach. Health Psychol Rev. 2016;10(3):297–312. 26262912 10.1080/17437199.2015.1077155 PMC4975080

[27] Adam D, Berschick J, Schiele JK, Bogdanski M, Schröter M, Steinmetz M, et al. Interventions to reduce stress and prevent burnout in healthcare professionals supported by digital applications: a scoping review. Front Public Health. 2023;11:1–10. 10.3389/fpubh.2023.1231266 PMC10630920 38026413

[28] Colquhoun HL, Squires JE, Kolehmainen N, Fraser C, Grimshaw JM. Methods for designing interventions to change healthcare professionals’ behaviour: a systematic review. Implement Sci. 2017;12(1):30. 28259168 10.1186/s13012-017-0560-5 PMC5336662

[29] Alarcon GM. A meta-analysis of burnout with job demands, resources, and attitudes. J Vocat Behav. 2011;79(2):549–62.

[30] Corry M, Clarke M, While AE, Lalor J. Developing complex interventions for nursing: a critical review of key guidelines. J Clin Nurs. 2013;22(17–18):2366–86. 23551526 10.1111/jocn.12173

[31] Sidani S, Braden CJ. Nursing and health interventions: design, evaluation, and implementation. Wiley; 2021.

[32] Glasgow RE, Vogt TM, Boles SM. Evaluating the public health impact of health promotion interventions: the RE-AIM framework. Am J Public Health. 1999;89(9):1322–7. 10474547 10.2105/ajph.89.9.1322 PMC1508772

[33] Michie S, Van Stralen MM, West R. The behaviour change wheel: a new method for characterising and designing behaviour change interventions. Implement Sci. 2011;6(1):42. 21513547 10.1186/1748-5908-6-42 PMC3096582

[34] Fletcher A, Jamal F, Moore G, Evans RE, Murphy S, Bonell C. Realist complex intervention science: applying realist principles across all phases of the medical research council framework for developing and evaluating complex interventions. Evaluation. 2016;22(3):286–303. 27478401 10.1177/1356389016652743 PMC4946011

[35] De Silva MJ, Breuer E, Lee L, Asher L, Chowdhary N, Lund C, et al. Theory of change: a theory-driven approach to enhance the medical research council’s framework for complex interventions. Trials. 2014;15(1):267. 24996765 10.1186/1745-6215-15-267 PMC4227087

[36] Höhmann U, Bartholomeyczik S. Komplexe Wirkungszusammenhänge in der Pflege erforschen: konzepte Statt rezepte. Pflege Gesellschaft. 2013;18(4):293–312.

[37] Wallner M, Mayer H, Adlbrecht L, Hoffmann AL, Fahsold A, Holle B, et al. Theory-based evaluation and programme theories in nursing: a discussion on the occasion of the updated medical research Council (MRC) framework. Int J Nurs Stud. 2023;140:104451. 36812849 10.1016/j.ijnurstu.2023.104451

