# Supplementary material for: Designing the LAGOM burnout prevention program: a collaborative approach with healthcare professionals using intervention mapping
Source: BMC Med Educ. 2025 Sep 16;25:1254. doi: 10.1186/s12909-025-07943-9 (PMC12439362; doi:10.1186/s12909-025-07943-9)
Supplement: Supplementary file 1 — Supplementary Material 1 [file 12909_2025_7943_MOESM1_ESM.docx]

## Supplement

**Supplement 1** Relevance and changeability of the environmental outcomes as assessed by the project´s advisory boards

| **Needs Assessment Category** | **Topic** | **Rele-vance** | **Change-ability** | **Sum 1:1.5** | **Inclusion based on cut-off basis and team discussion** |
| --- | --- | --- | --- | --- | --- |
| **Interpersonal: Teams** | Missing appreciation (by peers) | 3 | 3,4 | 8,1 | X |
|  | Language Barriers / Intercultural Challenges | 3 | 2,7 | 7,1 |  |
|  | Fluctuating teams - no emotional connection due to team rotations | 3 | 2,6 | 6,9 |  |
|  | Co-workers with unhealthy behavior (lack of role models) | 2 | 3,1 | 6,7 |  |
|  | Interruptions (relatives, phone calls) | 3 | 2,4 | 6,6 |  |
|  | Conflicts with the integration of leasing personnel | 2 | 2,8 | 6,2 | X |
|  | Lack of communication skills and methods/styles | 1 | 3,4 | 6,1 |  |
|  | Fear of communication overload (of being laughed at) | 1 | 3,3 | 5,9 |  |
|  | Not asking for help because of (assumed) overload of colleagues | 1 | 3,1 | 5,7 |  |
|  | Tensions between nurses and physicians | 1 | 2,8 | 5,3 |  |
|  | Conflicts due to generational differences | 1 | 2,7 | 5,1 |  |
| **Interpersonal: Leadership / Leadership Level** | Missing appreciation (by leaders) | 3 | 3,3 | 7,9 | X |
|  | Lacking feedback routines | 3 | 3,0 | 7,5 | X |
|  | Unhealthy role modelling | 1 | 2,7 | 5,1 |  |
| **Organisational: Psychological working conditions** | Increasing red-tape | 4 | 2,4 | 7,6 |  |
|  | Time pressure (no time to drink, eat, breaks) | 4 | 3,0 | 8,5 |  |
|  | Dealing with suffering/waiting and dying patients | 3 | 2,5 | 6,8 |  |
|  | Insufficient work-life balance | 3 | 3,0 | 7,5 |  |
|  | High workload = little time for primary nursing or medical activities | 2 | 2,8 | 6,3 |  |
|  | Insufficient onboarding / training (in terms of time and content) | 2 | 3,3 | 6,9 |  |
|  | Compliance to COVID-19 rules (missing team interaction, feeling of being left alone) | 1 | 2,8 | 5,3 |  |
|  | Inconsistent framework among teams e.g. for break routines | 1 | 3,0 | 5,5 |  |
|  | Peak of patient load | 1 | 2,3 | 4,5 |  |
|  | Problems associated to shift work | 1 | 2,0 | 4,0 |  |
|  | Job flexibility (e.g. going home when the child is ill) | 1 | 2,9 | 5,3 |  |
|  | Consideration of service plan proposals | 1 | 3,0 | 5,5 |  |
|  | Monotonous tasks | 1 | 2,7 | 5,1 |  |
|  | Optimizable "open ear" policy | 1 | 3,7 | 6,6 | X |
| **Organisational: Physical working conditions** | Unsatisfactory design/architecture of (break) rooms | 3 | 2,7 | 7,1 |  |
|  | Insufficient technical equipment (e.g. PC workstations) | 2 | 2,7 | 6,1 |  |
|  | Sensory overload / stressors (e.g. telephone, alarms) | 2 | 2,6 | 5,9 |  |
|  | Aggravated access to catering | 1 | 3,0 | 5,5 |  |
|  | Long distances (physical exhaustion) | 1 | 2,8 | 5,3 |  |
| **Organisational: Other** | Shortages of staff | 4 | 2,4 | 7,6 |  |
|  | Lacking gratification/appreciation/salary | 3 | 3,3 | 7,9 |  |
| **Society** | Lack of appreciation | 2 | 3,0 | 6,5 |  |
|  | Aggravated requirements due to the COVID-19 pandemic | 1 | 2,7 | 5,0 |  |
|  | Staff shortage | 1 | 2,4 | 4,6 |  |
|  | Conflicting staff key requirements | 1 | 3,0 | 5,5 |  |

**Supplement 2** List of personal and external determinants. Chosen determinants for further program development in bold

|  | **Provisional list** | **Additional literature research** |
| --- | --- | --- |
| Personal determinants | **Attitude**  Beliefs  **Self-efficacy**  **Knowledge**  **Outcome expectations**  Affective factors  Personal Ressources  **Awareness/Mindfulness** | Work addiction, workaholism  Loyality  Age/Generation |
| External determinants | **Reinforcement**  **Cues**  **Stigmatisation/Peer Pressure**  Work culture  Company culture  **Policies**  Leadership culture/ leadership behavior  Team culture | **Autonomy**  Workload  Shift work  Staffing  Social support  Accessibility  **Infrastructure / Accessability**  Not matching programs  Work unit, profession  Peer pressure  Management pressure  Financial resources  Planning uncertainty (Patient Peak during breaks)  Regulatories  Bullying |

**Supplement 3** Changing personal determinants: Final list of methods and strategies

| **Personal Determinant** | **Methods** | **Strategies** |
| --- | --- | --- |
| *Attitude* | Shifting perspective (Health Belief Model) | Documentation of thoughts/experience - Focusing on positive experiences daily/regularly/ appreciating them |
|  | Selfreflection (Transtheoretical Model, Social Cognitive Theory) | Self-test, reflection rounds, scales documentation, exchange in the group  Self-awareness (physically) experience, |
|  | Persuasive communication; Persuasion Communication Matrix, Elaboration Likelihood Model | Credible, comprehensible information in e.g. script / workbook, slide sets / presentations  Sources, Graphics |
|  | Active processing of information  (Persuasion Communication Matrix, Elaboration Likelihood Model) | Comic, video, discussion groups, internal development of content, self-reference |
|  | Repeated exposure (Theories of learning) | Posters, Questions, Homework / Mini-Impulses for every day / Booster Sessions (Follow Ups) |
| *Self-efficacy* | Direct Experience (Theory of Learning) | Exchange, recognition of experiments/insights/testing of content, exchange in the group and reinforcement, exercises for everyday life / pursuit of own goals |
|  | Modelling | Creating role models, trainers/managers as good role models (communication, etc.), demonstrating exercises/impulses |
|  | Guided practice | Live exercises, feedback, role plays, relaxation techniques |
|  | Self-Regulation | Goal setting  Commitment / difficult/availability - confidence/importance (scales), planning to overcome barriers, self-monitoring, securing reinforcements  maintaining social/external support |
|  | Planning Coping responses | Stress coping mechanisms, interruption techniques, etc. |
| *Knowledge* | Arguments / Information – Loss/gain frame | Positive/(negative) effects on health, rights - Psychoeducation |
|  | Tailoring (Trantheoretical model, Precaution adoption model) | Examples from everyday life, discussing patient cases |
|  | Discussion (Theories of information processing) | Interaction in group, small groups |
|  | Active learning (Persuasion communication matrix, elaboration likelihood, social cognitive theory) | Self-development of topics, active / independent elaboration, practice |
|  | Cues (theories of information processing) | Posters, calendars, reminders at work and in the course, |
| *Outcome expectations* | Shifting perspective (Health Belief Model) | *see attitudes* |
|  | Self-reevaluation / Self-reflection (Transtheoretical Model, Social Cognitive Theory) | Questionnaires, goal setting, scaling |
|  | Modelling | Role modelling on conducive behaviour, authentic, break behaviour |
| *Awareness / Mindfulness* | Self-reevaluation / Self-reflection/ Consciousness Raising (Transtheoretical Model, Social Cognitive Theory) | Self-test, meditation on body/thoughts/feelings, identification triggers |

**Supplement 4** Changing external determinants: Final list of methods and strategies

| **External Determinant** | **Methods** | **Strategies** |
| --- | --- | --- |
| *Reinforcement* | Modeling | Trainings on health-oriented leadership, commitment from higher management levels - common goals |
|  | Persuasive Communication *(see Attitude)* | Conversation skills (for employees) |
| *Cues* | Cues  Repeated exposure  *Also see attitude & knowledge* | Posters / desk calendars, reminders at work, mental health newsletters, inspirational things as a break screen (employees, role models), podcasts Charité (on mental health), food for thought, available yoga mats, water dispensers/snacks, juggling balls, movement stimulation via screen / reward / gamification (e.g. vending machine gives free coffee for squats), gong, reminder of mindfulness minute (on screen or on mobile phone, before team meeting) |
| *Stigma / Peer pressure* | Modeling | Team ambassadors (role models on several levels), buddy systems, video models, peers within the LAGOM program group |
|  | Stimulate communication and mobilizing social support (Transtheoretical Model) | Exchange groups (interprofesional) / Whatsapp groups, chat, support networks (for duty rosters, outage etc)  Mental Health Support Groups / Mental Health positive community |
|  | Non-judgemental group discussions (safe environment) | Role-Model Interviews (live or recorded)  Framework for group work – group rules for communication / joint work (confidentiality, etc.) |
| *Policies* | Model availablitiy | Organisational role models für mental health and Leitbild, Theory U |
|  | Advocacy | Press Conference, Information Event / Mental Health day / Mentoring Programs |
|  | Participatory Problem solving | Open-Ear day – moderated day on solutions/problems of the Charité and Immanuel hospital |
|  | Effective Change management | Chief Happiness Officer / Feel Good Manager, Mental Health professionals |
|  | Skills training (Theories of social networks Theory, Diffusion of Innovations of support needed and must intensively train  Theory, coalitions) | Mental Health Knowledge and Skills, Empathy – Communication training |
|  | Incentives | Merch, vouchers, discount offers, small gifts (also posters, postcards), catering for free |
| *Autonomy* | Modelling (Leads to Self-Efficacy, Needs Attitude Change)  *Also see reinforcement* | Leadership theories (transformational, cooperative, integral leadership) |
| *Infrastructure / Accessibility* | Technical Infrastrucutre and Support | IT support, platform for LAGOM etc. |
|  | Repeated exposure / information | Visibility offers, accessibility through appearance on the intranet, posters, emails, internal training fair |
|  | Rooms / Architecture | Suitable rooms with equipment (mats, beamer etc.), close to the workplace  Beautification of break rooms |

**Supplement 5** Interview guide

Introduction 2 Min

1. Introduction and gratitude for participation and consent.
2. Introduction to the topic: Survey background: Today, you are participating in a survey conducted during the preliminary phase of the planned ART study, which focuses on stress and burnout prevention. ART stands for Work-Related Resilience Training. We will be discussing your experiences and expectations regarding this topic.
3. There are no right or wrong answers here; we aim to learn about your personal opinions. As neutral scientific researchers, we want to understand your expectations, challenges, and the realities of medical work to design the planned program as effectively and tailored to your needs as possible.
4. We would like to make audio recordings of this interview. The collected data will solely be used to gather insights into expectations and the necessity of a stress management program and will, of course, be treated anonymously and confidentially. After 10 years, the data will be securely archived in compliance with data protection regulations. Do you agree to this?
5. The interview will take approximately 25 minutes.
6. Do you have any questions so far?

[Interviewer: start recording]

Background of the participants 15 Min

Before we ask you about potential requirements for future anti-stress programs, we would like to start with some more general questions about your experience with stress management.

- Please begin by introducing yourself briefly. How old are you, and what is your profession?
- What type of work structure do you operate in (inpatient, intensive care, outpatient, day clinic, functional area, outside of patient care)?
- Do you work shifts?
- About your workday:
  - Please describe a (typical) workday. How many hours do you usually work?
  - How much time do you typically have for rest or breaks during your workday?
  - How does your work influence your personal well-being or health?

Today's topic is stress.

- Please tell me more about yourself and your current experience with stress.
  - How would you rate your experience of stress on a scale of 1 to 10 (where 1 means very relaxed and 10 means extremely stressed)?
  - What do you consider to be the causes of the stress you experience?
  - What is your current energy level on a scale of 1 to 10 (where 1 means very exhausted and 10 means very energetic)?
- Have you ever taken steps to (better) manage your stress?
  - What did you try, and what has your experience been so far?
- [Optional Question] Are you familiar with or do you use digital tools (apps or web-based platforms, online training) for relaxation and stress management?
  - If yes: Which ones? Since when? How often?
  - What do you particularly like about them (features/content)?
  - Is there anything you don’t like or feel is missing?

Needs Fulfillment & Development – Expectations for Future Offerings 10 Min

Now that we have discussed your past experiences, we would like to turn to your expectations for future programs.

- How would a prevention program need to be designed for you to participate regularly (e.g., over an 8-week period)?
  - Content
  - Format/Time/Space/Duration
    - [Optional follow-up question] Would you prefer a program conducted in-person or (additionally) online/digitally?
- Should the program during practical courses include participants from different professional groups, or should it separate them (e.g., doctors/nurses)?
  - [Optional follow-up question] Would you prefer to know other participants (colleagues) in the training or for participation to remain anonymous (e.g., nurses from other institutions, as in an online format)?

Thank you very much. I have asked all of my questions. Is there anything else you would like to share with us?

Thank you for your participation!

[Interviewer: End recording]
